# Undifferentiated carcinoma of the pancreas with osteoclast-like giant cells, a two cases report

**DOI:** 10.1016/j.ijscr.2024.109419

**Published:** 2024-02-20

**Authors:** Maria Luisa Tambasco, Philippe Echelard, Florence Perrault, Rabia Temmar, Vincent Quoc-Huy Trinh, Yves Collin

**Affiliations:** a Faculty of Medicine and Health Sciences, University of Sherbrooke, Sherbrooke, Canada; b Department of Pathology, University of Sherbrooke, Sherbrooke, Canada; c Department of Pathology, Microbiology and Immunology, Vanderbilt University Medical Center, Nashville, United States; d Department of Surgery, University of Sherbrooke, Sherbrooke, Canada

**Keywords:** Pancreas, Osteoclast-like, Adenocarcinoma, Giant cells

## Abstract

**Introduction and importance:**

Fine needle aspiration is the standard method for the pathological evaluation of pancreatic masses. In the following context, rare variants of such masses might present a challenge. Our goal is to describe the clinical, cytological, and histological findings of two cases of undifferentiated carcinoma with osteoclast-like giant cells (UCOCGC) a rare variant of pancreatic ductal adenocarcinoma (PDAC).

**Case presentation:**

Two cases were identified. Cytological findings exhibit similarities between the two cases. One patient received multiple chemotherapy regimens and a surgery and recurred within three years of diagnosis, while the other succumbed to cholangitis resulting from hepatic metastases a year after their initial surgery.

**Discussion:**

UCOCGC is a rare variant of pancreatic cancer, characterized by a unique cytological aspect. Recognizing this variant is essential considering its distinct prognosis compared to usual pancreatic adenocarcinoma.

**Conclusion:**

We presented two cases of UCOCGC a rare pancreatic cancer variant, exposing diagnostic particularities and clinical evolution.

## Highlights

• Importance of differentiating UCOCGC a very rare form of cancer between other PDAC.
• Strongly suggested to generate future tests for patients with PDAC.
• Reduce the use of valuable resources and risks associated with irrelevant procedures.

## 1 Introduction

In this article, we present two cases of undifferentiated carcinoma with osteoclast-like giant cells (UCOCGC), a histological subtype of pancreatic ductal adenocarcinoma (PDAC). UCOCGC is a rare tumor often associated with intraductal papillary mucinous neoplasm and has a more favorable prognosis than usual PDAC.

## 2 Methods

We performed a review of the PDAC cases diagnosed at our institution over the past 14 years using the keywords “pancreatic adenocarcinoma with osteoclast-like giant-cells”. Our search yielded two cases harboring this diagnosis. Ethic approval and informed consent were obtained as per our institution guidelines. A review of the clinical files, cytological and histological slides was conducted. And the work has been reported in line with the SCARE criteria [37].

## 3 Results

**Fig. 1:**
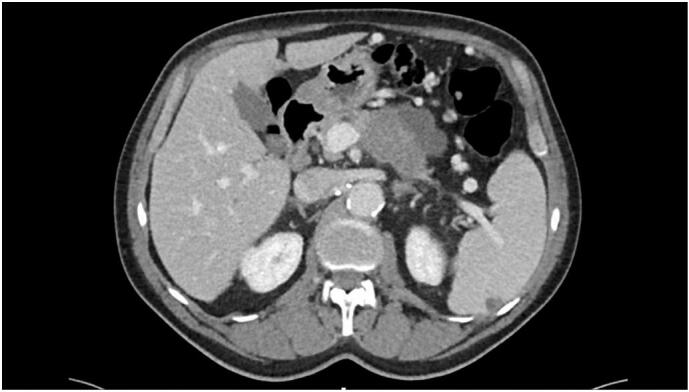
Patient 1 abdominal CT-scan.

**Fig. 2:**
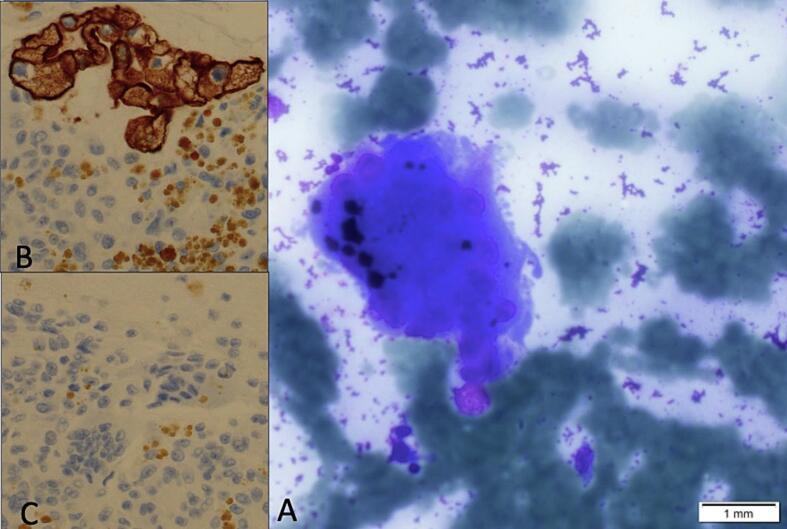
Case #1 shows atypical mononucleated cells (not shown here) and osteoclast giant cells with >20 nuclei without atypia (A: MGG ×40). Immunohistochemistry for CK-19 is positive in mononucleated tumor cells (B) and negative in giant cells and histiocytes (C).

First patient: A 71-years-old man presenting with symptoms of energy loss and abdominal pain, prompted evaluation at our institution in 2017. Imaging by ultrasound and abdominal CT-scan revealed a 6.4 × 3.5 cm mixed solid and cystic mass of the tail of the pancreas in proximity with the splenic artery and vein (Fig. 1). A first fine-needle aspiration (FNA) was performed in September 2017 during gastroscopic evaluation, and the results concurred with a high-grade intraductal papillary mucinous neoplasm (IPMN) with concomitant invasive adenocarcinoma. During the staging procedures, a TEP-Scan revealed the presence of two lung nodules suspicious of metastasis or lung primary. A biopsy of the lung nodules showed distinctive immunolabeling compared to the pancreas tumor. After discussion in multidisciplinary tumor board, the patient underwent gemcitabine and nab-paclitaxel treatment. After three months of this therapy, restaging showed a progression of the pulmonary lesions and a response of the pancreatic lesion. After a new tumor board discussion, it was opted to propose Carbo-Taxol treatment. This time, the lung site responded while the pancreatic tumor did not. During follow-up, facing atypical progression of the disease under different chemotherapy regimen, a new biopsy was deemed necessary to orient patient care. In July 2018, a second FNA of the pancreatic mass finally revealed the presence of an undifferentiated invasive component associated with osteoclast-like giant cells (Fig. 2). In this context, our multidisciplinary team suggested systemic palliative care, but the patient underwent a subtotal pancreatectomy with gastrectomy and splenectomy at another medical institution in August 2018. The pathologic report was consistent with a large tumoral mass with both solid and cystic components measuring 12 cm in the greatest dimension and a pathological stage pT3N0. The histological analysis was concordant with an adenocarcinoma comprising well-differentiated foci intertwined with undifferentiated foci having osteoclast-like giant cells intermixed. The patient underwent SBRT for the lung tumors ending in December 2018. In February 2020, a CT scan proved a peritoneal and hepatic recurrence, for which FOLFIRI was recommended and used until, the patient died from a car accident in December 2021.

**Fig. 3:**
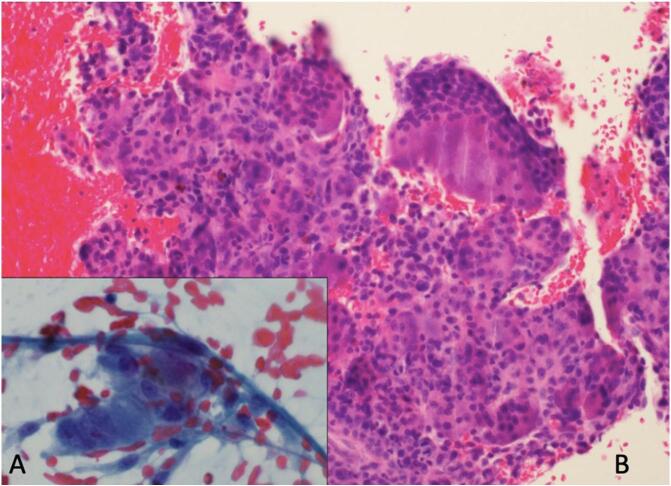
Case #2 shows atypical mononucleated cells on FNA (A: Papanicolaou x40). Cell block shows many osteoclast giant cells and histiocytes (B: H&E ×20).

**Fig. 4:**
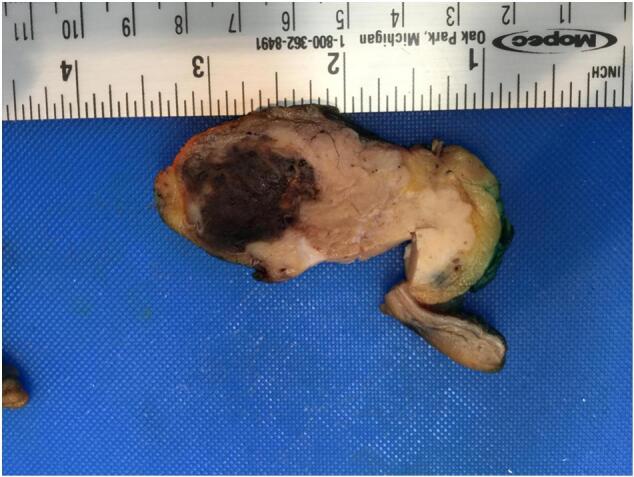
Case #2 Macroscopic specimen after Whipple procedure.

**Fig. 5:**
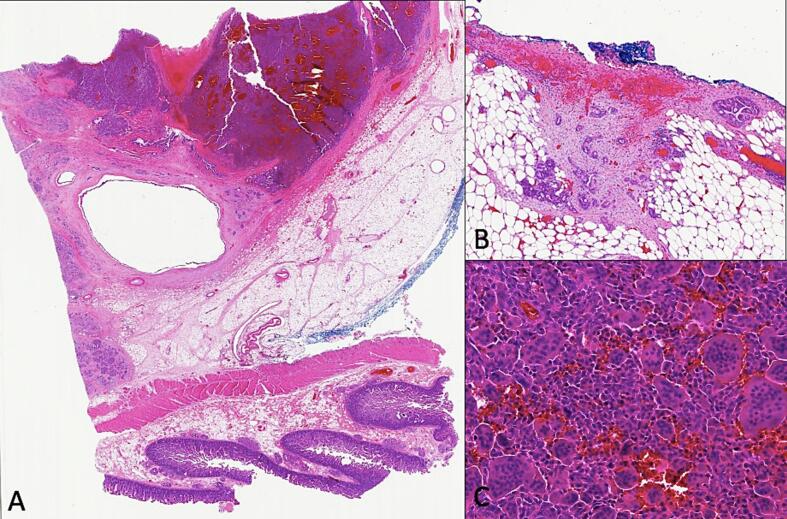
Case #2 shows a well delineated lesion of the head of the pancreas (A: H&E). Higher magnification revealed many histiocytes and osteoclast giant cells (C: H&E) along with usual pancreatic adenocarcinoma (B: H&E).

Second patient: In 2020, a 74-years-old man was evaluated for abdominal pain and jaundice. The gastroscopy performed showed a lesion of the head of the pancreas associated with distal choledochal sheathing. A FNA was conducted, revealing the presence of an undifferentiated invasive carcinoma coupled with osteoclast-like giant cells (Fig. 3). Immunolabeling showed positive for pancytokeratin AE1/AE3, CK8/18 p53 and CK7 in the carcinomatous cells while the mononucleated cells were CD68+. The patient underwent a Whipple procedure in March 2020. Macroscopic evaluation showed a 2.5 cm mass with a red/brown discoloration and hemorrhage (Fig. 4). The histological analysis confirmed the diagnosis of undifferentiated carcinoma with osteoclast-like giant cells (Fig. 5), and the pathological stage was pT2N1. After surgery, the patient was offered gemcitabine-based adjuvant chemotherapy, as he was deemed unfit for FOLFIRINOX. The patient went well at first, but unfortunately died in May 2021, from cholangitis secondary to a recurrence of the disease (hepatic metastases).

## 4 Discussion

PDAC is the most frequent pancreatic cancer [1]. Undifferentiated carcinoma (UC) is a rare, but very aggressive PDAC subtype known for displaying no clear differentiation within its cells [2]. Nonetheless, UC tissue may display non-neoplastic osteoclast-like giant cells (OCGC) generally resulting in a better prognosis and longer survival [3,4]. We present two rare cases of UCOCGC, with only 140 cases reported in the literature to our knowledge [[5], [6], [7], [8], [9], [10], [11], [12], [13], [14], [15], [16], [17], [18], [19], [20], [21], [22], [23], [24], [25], [26], [27], [28], [29], [30]]. UCOGGC is known to be rare, representing <2 % of all pancreatic malignant neoplasms [10]. The most recent WHO classification of digestive system tumors considers UCOCGC a distinct histological subtype of PDAC [31,32] also distinguishing it from UC without OCGC [1,3]. UCOCGC represents approximately 0.4 % of pancreatic tumors [5,6]. Both mesenchymal and epithelial origins are suggested for this tumor [4,31,33]. UCOCGC is composed of three main cell types that can appear under different proportions which consequently affect the prognosis: benign appearing osteoclast-like giant cells identical to osteoclasts found in bone, mononuclear tumor cells which can be spindled to epithelioid and pleomorphic giant cells suggesting a more malignant course [1,[3], [4], [5],^31^,^34^]. These findings can be readily identified morphologically in both fine needle aspiration and in core needle biopsies of the pancreas [6]. Interestingly, in our patient first patient, the first FNA did not show the undifferentiated aspect, most probably because of a simple sample bias (another portion of the tumor was sampled) or it could have developed during chemotherapy treatment before the second FNA.

Immunohistochemistry can also be used as an adjunct to the diagnosis of this tumor. The immunoprofile of osteoclast-like giant cells in UCOCGC suggests a mesenchymal origin as these cells are positive for CD68 and non-reactive to cytokeratin. OCGC's are also negative for anti-p53. The presence of pleomorphic giant cells supports the epithelial origin as they are reactive to cytokeratin and negative for CD68. They are also positive for anti-p53. As to the mononuclear sarcomatoid cells, they are reactive for both cytokeratin and CD68 and are also positive for anti-p53 [1,3,4,31,33,34]. Additionally, Ki-67 immunostain is useful, being weakly expressed by the osteoclast-like giant cells and highly expressed by the other two type of cells [[4], [5], [6],^31^]. Notably, vimentin strongly stains most lesion cells [1]. The presence of K-ras and p53 mutations are associated to increased component of undifferentiated carcinoma which can indicate a poorer prognostic [1,4]. Such results leads some authors into believing that the giant cells in UCOCGC are part of a reactive process not unlike giant cell tumor of the bones, while the pleomorphic giant cells are malignant progression of the sarcomatoid neoplastic cells, possibly representing entosis or other pathological processes [35]. Moreover, the elevated expression of the biomarker PD-L1 in OCGCs has been emphasized as a predictive indicator for the effectiveness of immune checkpoint inhibitors (ICIs), offering a potential avenue for future therapeutic interventions [3,33].

The relevance of properly diagnosing UCOCGC is its association with a protracted clinical course and much longer survival than usual PDAC with a five year survival rate of 59.1 % for UCOCGC versus 15.7 % for PDAC in one of the largest case series to date [5]. Without prior FNA, the five year survival rate is 57.5 % for UCOCGC versus 23 % for PDAC according to another study [6]. In this case, our first patient died 3 years after the initial surgery from an unrelated trigger that led to comfort care and the other patient passed away after 1 year of the first surgery from cholangitis secondary to hepatic metastases. Nevertheless, this raises the possibility that a different clinical approach might be warranted in these patients as at least a subset of them progresses particularly less aggressively than their classical counterparts. Furthermore, the pathophysiology underlying the presence of osteoclast-like giant cells have also been seen in other cancer sites and might underlie a particular interaction between tumor cells and the environment [36].

## 5 Conclusion

UCOCGC is a rare variant of PDAC that is increasingly recognized as a histological subtype associated with a favorable prognosis when correctly diagnosed. It is associated with distinct cytological and histological characteristics and its clinical course is deserving of further studies.

## Patient consent

Written informed consent was obtained from patients for scientific article and accompanying images publication. A copy of the written consent is available for review by the Editor-in-Chief of this journal on request.

## Ethical approval

Ethical approval was given the 21st of April 2020, by the Research Ethical Board of the “Centre Hospitalier Universitaire de Sherbrooke” at Sherbrooke with the Judgement's reference number 2020-3676.

## Funding

Funding comes from the Department of Surgery, 10.13039/100009874Université de Sherbrooke.

## Author contribution

Maria Luisa Tambasco, Philippe Echelard, Florence Perrault, Rabia Temmar, Vincent Quoc-Huy Trinh, Yves Collin, have contributed to study designing and data collection, data analysis and writing.

## Guarantor

Dr Collin.

## Research registration number

No Unique Identifying number or registration ID needed.

## Conflict of interest statement

The authors declare not to have conflict of interest.
